# Plasmonic Biosensing for Label-Free Detection of Two Hallmarks of Cancer Cells: Cell-Matrix Interaction and Cell Division

**DOI:** 10.3390/bios12090674

**Published:** 2022-08-24

**Authors:** Maria Carcelen, Veronica Vidal, Alfredo Franco, Marcos Gomez, Fernando Moreno, Jose L Fernandez-Luna

**Affiliations:** 1 Valdecilla Research Institute (IDIVAL), 39012 Santander, Spain; 2 Department of Applied Physics, Faculty of Sciences, University of Cantabria, 39013 Santander, Spain; 3 Genetics Department, Valdecilla University Hospital, 39008 Santander, Spain; 4 Department of Surgery, Valdecilla University Hospital, 39008 Santander, Spain

**Keywords:** cell cycle, cell adhesion, extracellular matrix, cancer cell, plasmonics

## Abstract

Two key features of cancer cells are sustained proliferation and invasion, which is preceded by a modification of the adhesion properties to the extracellular matrix. Currently, fluorescence-based techniques are mainly used to detect these processes, including flow cytometry and fluorescence resonance energy transfer (FRET) microscopy. We have previously described a simple, fast and label-free method based on a gold nanohole array biosensor to detect the spectral response of single cells, which is highly dependent on the actin cortex. Here we used this biosensor to study two cellular processes where configuration of the actin cortex plays an essential role: cell cycle and cell–matrix adhesion. Colorectal cancer cells were maintained in culture under different conditions to obtain cells stopped either in G0/G1 (resting cells/cells at the initial steps of cell growth) or G2 (cells undergoing division) phases of the cell cycle. Data from the nanohole array biosensor showed an ability to discriminate between both cell populations. Additionally, cancer cells were monitored with the biosensor during the first 60 min after cells were deposited onto a biosensor coated with fibronectin, an extracellular matrix protein. Spectral changes were detected in the first 20 min and increased over time as the cell–biosensor contact surface increased. Our data show that the nanohole array biosensor provides a label-free and real-time procedure to detect cells undergoing division or changes in cell–matrix interaction in both clinical and research settings.

## 1. Introduction

According to the World Health Organization, cancers caused 10 million deaths in 2020 and continue to be the leading cause of death in the world. The most prevalent ones are breast, lung, colorectal and prostate cancers, lung and colorectal cancers being the most common causes of cancer death [1].

Two biological features common to all cancers that hinder successful treatment and recovery of patients are their proliferation capacity and their ability to invade the surrounding tissue and to generate metastasis.

In contrast to normal cells, which control their growth by ensuring homeostasis, cancer cells are characterized by a sustained and dysregulated proliferation. Several mechanisms have been proposed to explain this and all of them activate intracellular signaling cascades that promote cell-cycle progression [2]. Furthermore, cancer cells not only sustain proliferation but also enable replicative immortality. Therapeutic strategies have been designed against these cancer-promoting mechanisms, such as inhibitors of the EGF receptor [3], cyclin-dependent kinase inhibitors and telomerase inhibitors, among others [2]. Despite the efficacy of some of them, controlling cancer cell proliferation continues to be a challenge in clinical research.

The invasiveness of many cancers is another challenge needed to be faced by researchers. The spreading of cancer cells allows the formation of new cancers in adjacent or distant tissues (metastasis), and this highly complicates surgical and pharmacological treatments. As a first step of the invasion process, cancer cells alter their adhesion properties, weakening cell–cell attachment and strengthening cell–matrix adhesions by means of adhesion molecules such as integrins and cadherins [4]. Cell adhesion has been studied by using techniques such as polyacrylamide traction force microscopy (PA-TFM) and three-dimensional traction force quantification (3D-TFM) [5]. Modifications of the actin cortex, which surrounds the periphery of the cell underneath the plasma membrane, facilitates cancer cell movement as it provides traction forces to the cell [6]. Proteins involved in cancer cell adhesion have been targeted by different therapeutic approaches, including the use of miRNAs [7,8] and blocking monoclonal antibodies [9,10], in an attempt to avoid cancer spreading.

Cell identification and biological processes can be revealed through changes in the optical properties of cells and, consequently, their refractive index (RI) is a relevant biophysical parameter. Different techniques have been applied to measure this parameter in different cell types and to study its spatial distribution within the cellular compartments [11,12].

Due to its high RI sensing sensitivity, the extraordinary optical transmission (EOT) phenomenon is the base of many biosensing tools for detecting small changes in the concentration of biomolecules dispersed in fluids [13,14]. EOT in metallic nanohole films is being explored as a tool for personalized medicine through the label-free analysis of live cells [15,16] because the EOT-based biosensors can reveal subtle changes in the interactions between the cells and the nanostructured films within the penetration depth of the excited plasmons [5,17,18]. The EOT-based techniques are suited to sense the outer layer of cells because the thickness of the plasmon penetration depth, for gold nanostructured films, is about 200 nm [19]. This region includes the actin cortex, which plays an important role in cellular processes such as cell division, cell migration and tumor cell invasion [20,21] by means of actin polymerization–depolymerization mechanisms.

The EOT-based biosensing techniques are an attractive label-free platform for healthcare settings because of the easy integration of their optical setup [22]. Thus, they are an alternative to more complex and fluorescence-dependent techniques to study cells and tissues. Recently, we described a plasmonic system based on a nanohole array biosensor to discriminate live single cancer cells from normal cells [23]. Other techniques, including confocal refractive index microscopy, terahertz spectroscopy and quantitative phase imaging [24,25,26], have been used for label-free imaging of living cells or cell-derived structures. The confocal imaging techniques have an advantage in investigating whole cells, which is due to their larger propagation depth, when there are no specific subcellular structures to target.

Our plasmonic system was tested with a number of colorectal cancer cell lines and primary colon cancer cells from surgical specimens. In all cases, the biosensor detected changes in the optical properties between cancer cells and normal cells that included skin fibroblasts, peripheral blood mononuclear cells and colon epithelial cells [23]. Moreover, we showed that the discrimination capacity of the system mostly relied on the configuration of the actin cortex, a complex network of polymeric actin assembled into long filaments located just underneath the plasma membrane, within the short penetration depth of the surface plasmon. This encouraged us to further study the capacity of the biosensor to detect cellular processes where the actin cortex plays a relevant role. Among them, there are two key processes typically associated with cancer development and progression: sustained cell proliferation and changes in cell–matrix interaction that precede migration of cancer cells.

Cancer cells have a dysregulated control of their cell cycle as a result of sustained proliferative signals and evasion of growth suppressors [2]. Thus, proliferation rates are higher in cancer cells than in normal cells. Flow cytometry is commonly used to analyze the different phases of the cell cycle during division, which requires the use of fluorescent DNA-binding dyes. The principle of this procedure is that dyes bind in proportion to the amount of DNA present in the cell. Therefore, during the S phase, the DNA content is duplicated with the corresponding increase in the fluorescence signal.

Healthy tissues control their homeostasis partly through cell–cell and cell–extracellular matrix (ECM) interactions and changes in these adhesion processes is a major hallmark of cancer. Moreover, epithelial–mesenchymal transition (EMT) is a program that allows epithelial cancer cells to change their adhesion properties, boosting their motility and invasive capacity. EMT is then considered the primary element driving cancer progression to metastasis [27]. Atomic force microscopy has been used in the study of cell–substrate and cell–cell interactions at the single-cell level [28] and permits the adhesion of living cells. Although it is a sensitive technique that can be applied even to characterize the interaction of single molecules, it requires complex instrumentation and it is time consuming. Moreover, the thermal drift in this system restricts adhesion studies to short contact times [29]. Alternatively, fluorescence resonance energy transfer (FRET) microscopy has been used to quantify several features of the cell–substrate interface including molecular changes induced by this interaction [30] and to study cell migration and adhesion by using fluorescent probes [31].

Here we proved the capacity of a nanohole array biosensor as an alternative easy-to-implement and label-free way to detect the interaction of cancer cells with the ECM and to discriminate between cell cycle phases G0/G1, which precedes DNA synthesis (S phase), and G2, where the DNA has been duplicated and cells initiate mitotic division. This biosensing method could be complementary to other systems able to study these cellular processes at the molecular level.

## 2. Materials and Methods

### 2.1. Optical Biosensor

The optical system and the configuration of the nanohole array biosensor have been extensively described elsewhere [23]. Briefly, the biosensor is composed of a nanostructured gold film deposited on a glass slide coated with a Ti film. The nanostructures are constituted by a square periodic array of circular nanoholes. The spectral shift (∆λ) of the light transmitted through the biosensor greatly depends on the refractive index (RI) of the material placed within 200 nm above the metallic surface, a region where the electric field is enhanced at plasmonic resonance conditions. The optical sensitivity of this spectral biosensor is in the order of 400 nm/RIU for resonant wavelengths around 750 nm. Its design was guided by simulations performed through Lumerical software (Ansys Inc., Canonsburg, PA, USA) and it was fabricated by e-beam lithography in the CIC nanoGUNE Research Center (Donostia, Spain). The optical system is a modified version of a motorized upright Eclipse Ni bright transmission microscope (Nikon Co., Tokio, Japan) coupled to an Andor Shamrock 303-i spectrograph (Oxford Instruments PLC, Oxfordshire, UK). We randomly focus individual cells within the nanostructured surface for RI measuring.

### 2.2. Cell Lines

Colorectal cancer cell lines CaCo2, HCT116 and SW480 were acquired from ATCC (Manassas, VA, USA). All cells are adherent with epithelial morphology. CaCo2 cells were cultured in Dulbecco’s Modified Eagle Medium (DMEM) (Biowest, Nuaillé, France) supplemented with 20% fetal bovine serum (FBS) (GE Healthcare-Hyclone, Chicago, IL, USA); HCT116 cells were cultured in McCoy’s 5A (Biowest, Nuaillé, France) supplemented with 10% FBS and SW480 cells in L-15 medium (ATCC) with 10% FBS. A mixture of antibiotics including 100 U/mL penicillin and 100 μg/mL streptomycin (Lonza, Basel, Switzerland) was added to all cell cultures. Cells were incubated at 37 °C and 5% CO_2_ in a humidified incubator (HeraCell, Heraeus, Hanau, Germany).

### 2.3. Cell Cycle Synchronization

Cancer cells were synchronized in the G0/G1 phase by incubating them in the presence of 10–12.5 µM lovastatin (Sigma Aldrich, St. Louis, MO, USA) for 24 h. Synchronization in the G2 phase was achieved with the addition of 0.05 µg/mL colchicine (Thermo Fisher Scientific, Waltham, MA, USA) to cell cultures for 24 h. Cells were then collected in phosphate buffered saline (Sigma Aldrich) for further analyses.

### 2.4. Fluorescence Activated Cell Sorting (FACS)

For cell-cycle analyses, cells were collected and permeabilized with 70% ethanol for 30 min. Cells were washed and incubated with 50 μg/mL propidium iodide (Sigma Aldrich) and 100 μg/mL ribonuclease A (Sigma Aldrich) as described previously [32]. Samples were kept in the dark at 4 °C until use. Flow cytometry was performed in a FACsAria II cytometer (BD Biosciences, San Jose, CA, USA).

### 2.5. Plasma Membrane Staining

CaCo2 cells were stained by using cell mask green plasma membrane stain (Thermo Fisher Scientific) following the manufacturer’s recommendations. Images of stained cells were obtained with a A1R spectral scanning confocal microscope (Nikon Co.) that incorporates a total internal reflection fluorescence (TIRF) module.

### 2.6. Optical Measurements with the Biosensor

To study the cell–matrix interactions 2.5 × 10^5^ cells were resuspended in culture medium and deposited on the nanohole array biosensor pretreated with fibronectin as an ECM protein [33] and maintained at 37 °C by using a homemade heating chamber. A solution of 10 μg/mL fibronectin (Sigma Aldrich) was used for coating the gold surface of the biosensor for 1h at room temperature. Experiments were performed in triplicate. For each experiment, 10 cells were randomly selected and the spectrum of the transmitted light was sequentially measured every 20 min.

For cell-cycle analyses, cells synchronized in G0/G1 phase or G2 phase were deposited onto the biosensor and 25 cells (in triplicate) were subjected to optical transmission measurement in each condition.

All measurements were made under the same experimental conditions, using phosphate-buffered saline as a medium. First, a full scanning of the fibronectin-coated biosensor was performed to assure its homogeneity [34]. Second, a reference spectrum was taken by selecting an area without cells. Finally, a spectrum of the cell was taken. Δλ was obtained from the comparison of both spectra [23].

### 2.7. Statistical Analyses

Thresholds for Δλ were set following the Youden index optimization criteria [35]. Data are represented as mean ± standard deviation of three independent experiments. Differences between groups were tested by Student’s *t*-test. The significance level was set at *p* < 0.05. All statistical analyses were performed using GraphPad Prism 5.0 (GraphPad Software, Inc., San Diego, CA, USA).

## 3. Results and Discussion

### 3.1. The Biosensor Is Able to Detect Different Phases of the Cell Cycle

**Figure 1:**
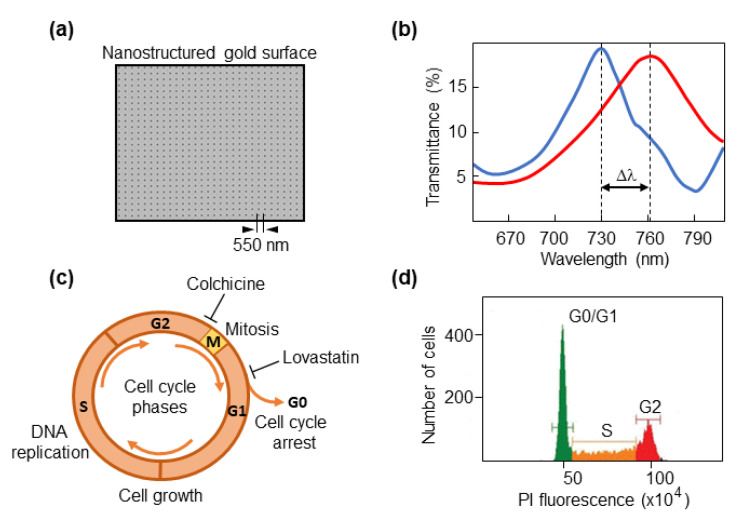
Cell-cycle phases and synchronization strategies. (**a**) Micrograph showing the sensing nanostructured area. The period of 550 nm is indicated. (**b**) An example of a spectral profile. Δλ corresponds to the spectral shift when a cell is located on the biosensor area. (**c**) Schematic representation of phases of the cell cycle. G0 phase, quiescent/resting state; G1 phase, cell growth starts by synthesizing proteins; S phase, DNA is duplicated; G2 phase, cells prepare to enter mitotic division. Colchicine was used to synchronize cells in G2 phase, and lovastatin was used for synchronization in G1. (**d**) A typical graphical representation of a cell cycle analysis by flow cytometry, that serves to verify the efficiency of synchronization. The X-axis shows the fluorescence intensity of propidium iodide (PI) in arbitrary units.

We used our previous optical setup with the plasmonic nanohole biosensor (Figure 1a) to detect spectral shifts in cells (Figure 1b). No significant changes in transmission were detected in the measurements performed in this work. To test the capacity of the biosensor to discriminate between different phases of the cell cycle, cancer cells were synchronized either in G0/G1, which precedes DNA synthesis, or the G2 that goes after DNA has been duplicated and precedes mitotic division (Figure 1c). Colorectal cancer cells were cultured with lovastatin, which mediates inhibition of DNA replication and arrests cells in early G1 phase by disrupting the proteasome pathway [36,37]. Thus, most cells would be either in the G0 (quiescent/resting state) or G1 phase. As we used a fluorescent DNA dye to measure DNA content in a flow cytometer, resting G0 cells and cells in G1, an interphase state that represents the first step of cell growth, appear in a single peak (DNA content 2N, diploid) followed by a flat S phase in the FACS profile where the DNA is being duplicated (Figure 1d).

**Figure 2:**
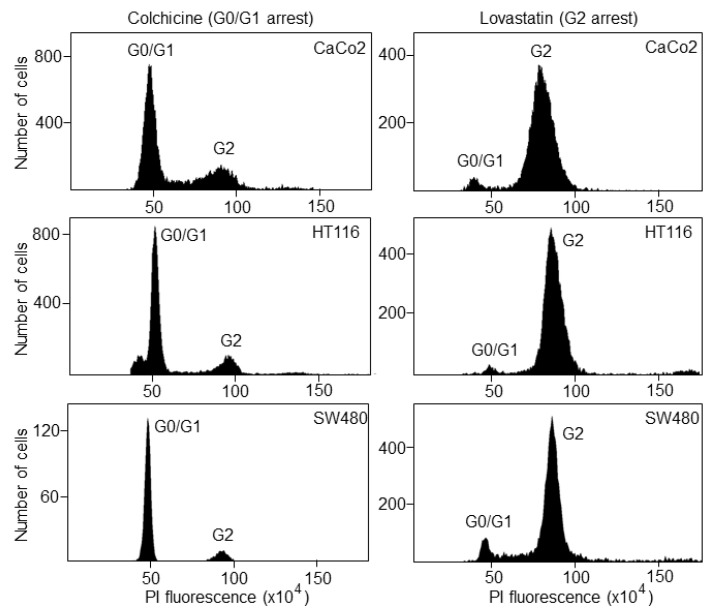
Cell-cycle synchronization of colorectal cancer cell lines. CaCo2, HCT116 and SW480 cells were treated with lovastatin or colchicine and then incubated with propidium iodide for cell-cycle analysis by FACS. G0/G1 and G2 represent the cell cycle phases before and after DNA synthesis, respectively.

Once the S phase has finished, the DNA content is 4N (tetraploid), which corresponds to the G2 phase. Following treatment with lovastatin or colchicine, about 80% of all cancer cell lines were arrested in the G0/G1 or G2 phase, respectively, as determined by FACS analyses (Figure 2).

**Figure 3:**
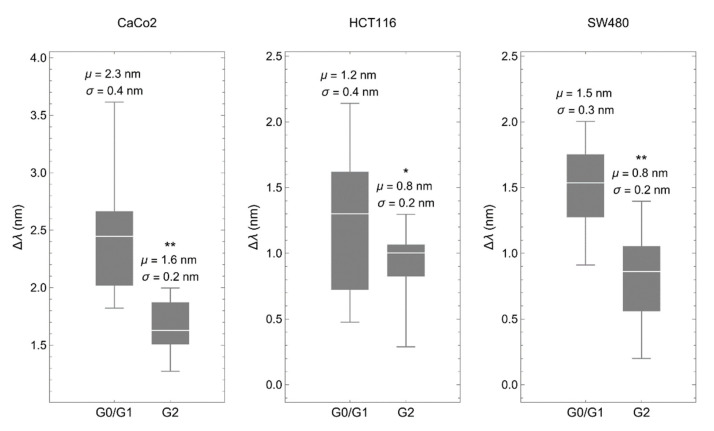
The biosensor discriminates between cell-cycle phases. Spectral shift (Δλ) comparing colorectal cancer cells synchronized in G0/G1 with the same cells synchronized in G2. The synchronization procedure was the same as described above. μ represents the mean and σ the standard deviation. * *p* < 0.05, ** *p* < 0.001.

Then, we deposited synchronized cells onto the biosensor to determine the spectral shift of the transmitted light, which depends linearly on the refractive index of the material placed within the 200 nm above the metallic surface, a region with the strongest electric field caused by plasmonic resonance [23]. Optical measurements obtained in all three cancer cell lines gave similar results. Interestingly, cells in G0/G1 showed RI values significantly higher than cells in G2 (Figure 3).

There are two arguments to discard that the compounds used for cell-cycle arrest affect the RI of the analyzed cells. Lovastatin inhibits the proteasome, which is present in the cytoplasm, mainly the pericentrosomal area and in the nucleus [38], and colchicine binds to soluble tubulin in the cytoplasm to prevent microtubule elongation [39]. Thus, neither one specifically targets cellular structures within the plasmonic area. In addition, the RI of colchicine is higher than that of lovastatin (1.58 vs. 1.53). If the compounds would have a significant contribution, plots in Figure 3 would be the other way around, with higher values of ∆λ in G2.

As we previously described, differences in RI between cancer and normal cells are mainly influenced by the actin cortex, which lies within the short penetration depth of the surface plasmon generated in the biosensor. Changes in thickness and density of the actin cortex may explain these differences [23]. Consistent with this, it has been shown that the thickness of the actin cortex is lower in mitosis compared to interphase in cancer cells, which inversely correlates with an increase in cortex tension [40].

There are different procedures that enable discrimination between G0 cells and G1 cells by FACS, which require fixation and permeabilization [32], two processes that have been shown to change actin cortex organization [41]. Thus, although we are aware that our experimental approach does not allow us to distinguish these two phases of the cell cycle, the biosensor is intended to be used in live cells without the need of disrupting manipulations. Considering that propidium iodide single-staining FACS is routinely used not only in experimental research but also in clinical settings, providing information to evaluate tumor aggressiveness and prognosis [42], the plasmonic biosensor could have some advantages over this standard technique.

Additionally, once the optical measurements have been determined, cells with RI values corresponding to G0/G1 could be isolated by using micro-pipetting cell pickers, successfully used to obtain single cells [43], for further studies that provide a more accurate identification of their cell-cycle status.

### 3.2. The Cancer Cell–Matrix Interaction Can Be Monitored by the Optical Biosensor

**Figure 4:**
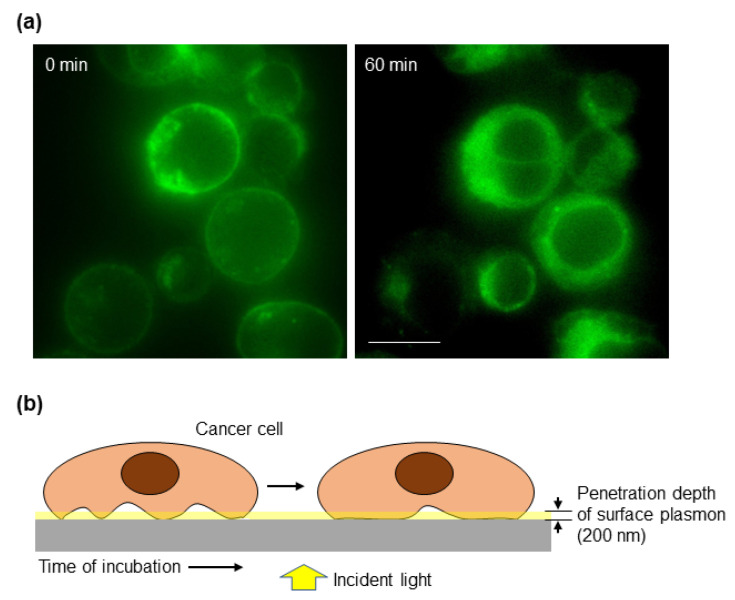
Cell–matrix interaction dynamics. (**a**) CaCo2 cells were labeled with a cell membrane fluorescent dye and immediately placed on fibronectin-coated slides and analyzed under a TIRF microscope. The critical angle was maintained until the end of the 60 min observation period. Images were acquired with a 100× immersion objective. Scale bar: 10 μm. (**b**) Schematic representation of the cell–substrate interaction over time. The penetration depth of surface plasmon, about 200 nm, is indicated to highlight the part of the cell that is detected by the biosensor.

Another key feature of cancer cells is their ability to modify their cell–cell and cell–ECM interactions to promote invasion of the surrounding tissue and eventually to establish a distant metastasis [2]. Typically, the actin cortex is thicker in rounded cells than in spread cells. Using high-resolution imaging, it has been shown that cortex thickness is reduced at high spread areas, which is also found during monocytic differentiation that takes place concurrent to cell spreading [44]. Based on this and taking into account the key contribution of the actin cortex to the spectral changes detected by the biosensor, we studied whether our optical system could monitor the process of cell attachment to a substrate. To have a more physiologically relevant model, the biosensor was coated with fibronectin, a ubiquitous ECM protein. Cells deposited onto the substrate increase their contacts (focal adhesions) with the ECM generating tension forces that will allow cell spreading. These contacts may be transmitted from the periphery, where they allow the cell to be more sensitive to changes in the microenvironment [45], to the center of the cell, as it can be deduced from the TIRF images (Figure 4a) and as it has also been previously modeled [46]. Cancer cells were stained with a fluorescent membrane marker and deposited on a fibronectin-coated slide for TIRF microscopy, which has been widely used for studying cell adhesion and the dynamics of the outer layers of the cell [47]. The penetration depth of TIRF microscopy depends on the angle of illumination resulting in a range of depths that typically vary from 70 to 200 nm [47]. Then, TIRF microscopy allowed us to focus on the first 200 nm above the surface, which is the penetration depth of the surface plasmon detected by the biosensor. As shown in Figure 4a, 60 min after cells were deposited onto the slide, there were more cell cortexes within the plasmonic area as determined by an increase in the fluorescent signal. Thus, we envision a model where the size of the cell cortex under the plasmon influence increases over time (Figure 4b).

**Figure 5:**
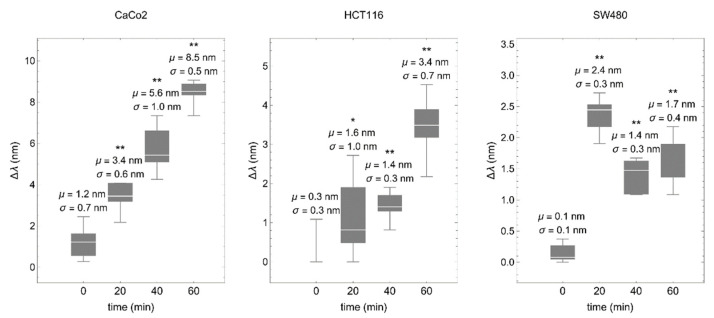
The biosensor detects cell–matrix interactions in real time. Colorectal cancer cells were deposited onto the fibronectin-coated biosensor and values of the spectral shift (∆λ) were obtained every 20 min. μ represents the mean and σ the standard deviation. * *p* < 0.05, ** *p* < 0.001.

Colorectal cancer cells were deposited on a fibronectin-coated biosensor and values of the spectral shift (proportional to the mean effective RI) were taken every 20 min. Consistently, in all cases, RI was increased over time, although the time-course profile was different in each cell line, most likely reflecting different cell adhesion dynamics (Figure 5).

Coupling of the cell to the ECM is due to focal and fibrilar adhesions that connect the actin cytoskeleton to the ECM through adhesion receptors from the integrin family, including the fibronectin receptor. At the cellular level, adhesion molecules do not function just as molecular glue but are highly dynamic. The formation of adhesive bonds depends on forces both self-generated internally by the cells or stemmed from cell–cell contact or inhomogeneous substrates [48]. These forces may change over time. For instance, the cytoskeletal protein tensin that along with the fibronectin receptor form cell–matrix fibrilar adhesions, has a highly dynamic behavior over time, moving either along the cell margins or in more central locations within a time period of 50 min [49]. These changes in the cellular location of adhesion molecules modify the cell–matrix contacts and may explain the variation of the spectral response we detect over time in the different cell lines as our system detects nanometric fluctuations in the cell–matrix boundary.

Adhesion depends on interaction between cellular receptors including integrins and cadherins, and ligands on the ECM. This interaction triggers cell pathways that promote cell spreading over the substrate and strengthening of cell–substrate adhesions [50]. Thus, the composition and density of adhesion receptors on the cell surface, as well as the strength of receptor-ligand bonds and the type of ECM present, may lead to different adhesion dynamics. Moreover, adhesion dynamics involve changes in the cellular traction forces, generated during rearrangement of ECM ligands, in the process of forming stable cell–substrate adhesion contacts [30]. In line with this, here we used fibronectin as the ECM protein, and differences in the expression of fibronectin receptors have been described among colorectal cancer cell lines [51,52].

Overall, our study addresses the use of a label-free biosensor for real-time detection of changes in cell cycle status or cell–matrix adhesion of live cancer cells, which could provide information about cancer aggressiveness and prognosis.

## 4. Conclusions

Sustained proliferative signaling and activation of invasion and metastatic programs are two major hallmarks of cancer development and progression that have attracted interest from researchers to develop novel detection and therapeutic strategies. Flow cytometry is widely recognized as the gold standard for quantification of cellular populations and cell-cycle analyses and represents a key tool in cancer diagnosis. Ki-67 immunohistochemical staining is also an indispensable nuclear antigen to study cell proliferation in cancer tissue sections. However, both techniques need to pretreat biological samples and use staining protocols to obtain accurate results. We have used a real-time, label-free optical biosensor to distinguish between resting cells or cells initiating proliferation and proliferating cells entering mitotic division. In addition, we also proved that this optical system can be used to study cell–matrix interactions. Cancer invasion is associated with a profound reorganization of the cytoskeleton to weaken cell–cell attachment and strengthen cell–matrix adhesions. Interest has been gained to develop strategies able to elucidate the adhesion properties of cancer cells at the single cell level over time to find markers that anticipate cancer invasion. Confocal refractive index microscopy based on the polarization-sensitive absorption of graphene [26] has also been used for the label-free imaging of living cells when there are no specific subcellular structures to target.

Overall, we propose our optical biosensor as an easy-to-implement approach that focusses on the cell cortex to study two key features of cancer cells, in both research and clinical settings.

## Data Availability

Not applicable.

## References

[1] Ferlay J., Colombet M., Soerjomataram I., Parkin D.M., Piñeros M., Znaor A., Bray F. (2021). Cancer statistics for the year 2020: An overview. Int. J. Cancer.

[2] Hanahan D., Weinberg R.A. (2011). Hallmarks of cancer: The next generation. Cell.

[3] Dutta P.R., Maity A. (2007). Cellular responses to EGFR inhibitors and their relevance to cancer therapy. Cancer Lett..

[4] Cavallaro U., Christofori G. (2001). Cell adhesion in tumor invasion and metastasis: Loss of the glue is not enough. Biochim. Biophys. Acta.

[5] Tu L., Li X., Bian S., Yu Y., Li J., Huang L., Liu P., Wu Q., Wang W. (2017). Label-free and real-time monitoring of single cell attachment on template-stripped plasmonic nano-holes. Sci. Rep..

[6] Schaks M., Giannone G., Rottner K. (2019). Actin dynamics in cell migration. Essays Biochem..

[7] Jurmeister S., Baumann M., Balwierz A., Keklikoglou I., Ward A., Uhlmann S., Zhang J.D., Wiemann S., Sahin Ö. (2012). MicroRNA-200c Represses Migration and Invasion of Breast Cancer Cells by Targeting Actin-Regulatory Proteins FHOD1 and PPM1F. Mol. Cell. Biol..

[8] Hu X., Guo J., Zheng L., Li C., Zheng T.M., Tanyi J.L., Liang S., Benedetto C., Mitidieri M., Katsaros D. (2013). The Heterochronic microRNA let-7 Inhibits Cell Motility by Regulating the Genes in the Actin Cytoskeleton Pathway in Breast Cancer. Mol. Cancer Res..

[9] Xie B., Priest A.V., Maker A., Gumbiner B., Sivasankar S. (2022). Molecular mechanisms for strengthening E-cadherin adhesion using a monoclonal antibody. Biophys. J..

[10] Li G., Suzuki H., Asano T., Tanaka T., Suzuki H., Kaneko M.K., Kato Y. (2022). Development of a Novel Anti-EpCAM Monoclonal Antibody for Various Applications. Antibodies.

[11] Liu P.Y., Chin L.K., Ser W., Chen H.F., Hsieh C.-M., Lee C.-H., Sung K.-B., Ayi T.C., Yap P.H., Liedberg B. (2016). Cell refractive index for cell biology and disease diagnosis: Past, present and future. Lab Chip.

[12] Gul B., Ashraf S., Khan S., Nisar H., Ahmad I. (2021). Cell refractive index: Models, insights, applications and future perspectives. Photodiagnosis Photodyn. Ther..

[13] Gordon R., Sinton D., Kavanagh K.L., Brolo A.G. (2008). A New Generation of Sensors Based on Extraordinary Optical Transmission. Accounts Chem. Res..

[14] Escobedo C. (2013). On-chip nanohole array based sensing: A review. Lab Chip.

[15] Li X., Soler M., Özdemir C.I., Belushkin A., Yesilköy F., Altug H. (2017). Plasmonic nanohole array biosensor for label-free and real-time analysis of live cell secretion. Lab Chip.

[16] Soler M., Lozano O.C., Estevez M.-C., Lechuga L.M. (2020). Nanophotonic Biosensors: Driving Personalized Medicine. Opt. Photon News.

[17] Zhou X., Yang Y., Wang S., Liu X. (2019). Surface Plasmon Resonance Microscopy: From Single-Molecule Sensing to Single-Cell Imaging. Angew. Chem. Int. Ed..

[18] Wang W., Wang S., Liu Q., Wu J., Tao N. (2012). Mapping Single-Cell–Substrate Interactions by Surface Plasmon Resonance Microscopy. Langmuir.

[19] Barreda .I., Otaduy D., Martín-Rodríguez R., Merino S., Fernández-Luna J.L., González F., Moreno F. (2018). Electromagnetic behavior of dielectric objects on metallic periodically nanostructured substrates. Opt. Express.

[20] Olson M.F., Sahai E. (2009). The actin cytoskeleton in cancer cell motility. Clin. Exp. Metastasis.

[21] Romet-Lemonne G., Jégou A. (2013). Mechanotransduction down to individual actin filaments. Eur. J. Cell Biol..

[22] Couture M., Live L.S., Dhawan A., Masson J.-F. (2012). EOT or Kretschmann configuration? Comparative study of the plasmonic modes in gold nanohole arrays. Analyst.

[23] Franco A., Vidal V., Gómez M., Gutiérrez O., Martino M., González F., Moreno F., Fernández-Luna J.L. (2022). A label-free optical system with a nanohole array biosensor for discriminating live single cancer cells from normal cells. Nanophotonics.

[24] Cao Y., Chen J., Zhang G., Fan S., Ge W., Hu W., Huang P., Hou D., Zheng S. (2021). Characterization and discrimination of human colorectal cancer cells using terahertz spectroscopy. Spectrochim. Acta Part A Mol. Biomol. Spectrosc..

[25] Zadka Ł., Buzalewicz I., Ulatowska-Jarża A., Rusak A., Kochel M., Ceremuga I., Dzięgiel P. (2021). Label-Free Quantitative Phase Imaging Reveals Spatial Heterogeneity of Extracellular Vesicles in Select Colon Disorders. Am. J. Pathol..

[26] Sun L., Wang Y., Zhang H., Min C., Zhang Y., Zhang C., Xin Z., Zhu S., Yang Y., Burge R.E. (2020). Graphene-based confocal refractive index microscopy for label-free differentiation of living epithelial and mesenchymal cells. ACS Sensors.

[27] Pastushenko I., Brisebarre A., Sifrim A., Fioramonti M., Revenco T., Boumahdi S., Van Keymeulen A., Brown D., Moers V., Lemaire S. (2018). Identification of the tumour transition states occurring during EMT. Nature.

[28] Moreno-Cencerrado A., Iturri J., Pecorari I., Vivanco M.D., Sbaizero O., Toca-Herrera J.L. (2017). Investigating cell-substrate and cell-cell interactions by means of single-cell-probe force spectroscopy. Microsc. Res. Tech..

[29] Helenius J., Heisenberg C.-P., Gaub H.E., Muller D.J. (2008). Single-cell force spectroscopy. J. Cell Sci..

[30] Huebsch N.D., Mooney D. (2007). Fluorescent resonance energy transfer: A tool for probing molecular cell–biomaterial interactions in three dimensions. Biomaterials.

[31] Kemp-O’Brien K., Parsons M. (2013). Using FRET to analyse signals controlling cell adhesion and migration. J. Microsc..

[32] Kim K.H., Sederstrom J.M. (2015). Assaying Cell Cycle Status Using Flow Cytometry. Curr. Protoc. Mol. Biol..

[33] Pankov R., Yamada K.M. (2002). Fibronectin at a glance. J. Cell Sci..

[34] Franco A., Otaduy D., Barreda A., Fernández-Luna J., Merino S., González F., Moreno F. (2019). Optical inspection of manufactured nanohole arrays to bridge the lab-industry gap. Opt. Laser Technol..

[35] PMartínez-Camblor P., Pardo-Fernandez J.C. (2019). The Youden Index in the Generalized Receiver Operating Characteristic Curve Context. Int. J. Biostat..

[36] JavanMoghadam-Kamrani S., Keyomarsi K. (2008). Synchronization of the cell cycle using Lovastatin. Cell Cycle.

[37] Rao P.S., Rao U.S. (2020). Statins decrease the expression of c-Myc protein in cancer cell lines. Mol. Cell. Biochem..

[38] Wójcik C., DeMartino G.N. (2003). Intracellular localization of proteasomes. Int. J. Biochem. Cell Biol..

[39] Leung Y.Y., Li L., Kraus V.B. (2015). Colchicine: Update on mechanisms of action and therapeutic uses. Semin. Arthritis Rheum..

[40] Chugh P., Clark A.G., Smith M.B., Cassani D.A.D., Dierkes K., Ragab A., Roux P.P., Charras G., Salbreux G., Paluch E. (2017). Actin cortex architecture regulates cell surface tension. Nat. Cell Biol..

[41] Charras G.T., Hu C.-K., Coughlin M., Mitchison T.J. (2006). Reassembly of contractile actin cortex in cell blebs. J. Cell Biol..

[42] Golias C., Charalabopoulos A. (2004). Cell proliferation and cell cycle control: A mini review. Int. J. Clin. Pract..

[43] Ungai-Salánki R., Gerecsei T., Fürjes P., Orgovan N., Sándor N., Holczer E.G., Horvath R., Szabó B. (2016). Automated single cell isolation from suspension with computer vision. Sci. Rep..

[44] Kumar R., Saha S., Sinha B. (2019). Cell spread area and traction forces determine myosin-II-based cortex thickness regulation. Biochim. Biophys. Acta.

[45] Stolarska M.A., Rammohan A.R. (2017). Center or periphery? Modeling the effects of focal adhesion placement during cell spreading. PLoS ONE.

[46] Wang N., Tytell J.D., Ingber D.E. (2009). Mechanotransduction at a distance: Mechanically coupling the extracellular matrix with the nucleus. Nat. Rev. Mol. Cell Biol..

[47] Grigoriev I., Akhmanova A. (2010). Microtubule Dynamics at the Cell Cortex Probed by TIRF Microscopy. Methods Cell Biol..

[48] Zieber F., Aranson I.S. (2016). Computational approaches to substrate-based cell motility. Comput. Mater..

[49] Zamir E., Katz M., Posen Y., Erez N., Yamada K., Katz B.-Z., Lin S., Lin D.C., Bershadsky A., Kam Z. (2000). Dynamics and segregation of cell–matrix adhesions in cultured fibroblasts. Nat. Cell Biol..

[50] Chalut K.J., Paluch E.K. (2016). The Actin Cortex: A Bridge between Cell Shape and Function. Dev. Cell.

[51] Kuwada S.K., Li X. (2000). Integrin α5/β1 Mediates Fibronectin-dependent Epithelial Cell Proliferation through Epidermal Growth Factor Receptor Activation. Mol. Biol. Cell.

[52] Yang G.-Y., Xu K.-S., Pan Z.-Q., Zhang Z.-Y., Mi Y.-T., Wang J.-S., Chen R., Niu J. (2008). Integrin alphavbeta6 mediates the potential for colon cancer cells to colonize in and metastasize to the liver. Cancer Sci..

